# Supplementary material for: Incidence and risk factors of perioperative respiratory adverse events in pediatric surgical patients: Development and validation of a predictive model in Brazil
Source: PLoS One. 2026 Apr 21;21(4):e0347477. doi: 10.1371/journal.pone.0347477 (PMC13098903; doi:10.1371/journal.pone.0347477)
Supplement: S1 Table — (DOCX) [file pone.0347477.s001.docx]

**Supporting information**

**S.1 Table -** Detailed variables and outcomes description.

| **Respiratory Adverse events** | *Oxygen desaturation*: pulse oximetry < 90% for at least 1 minute;(1, 2) *stridor*: usually a high-pitched sound produced by the rapid, turbulent flow of air through a narrowed segment of the large airways, mostly inspiratory and almost always heard without a stethoscope;(2) *bronchospasm*: new wheezing detected on pulmonary auscultation and treated with bronchodilators;(3, 4) *laryngospasm*: occlusion of the glottis by the laryngeal muscles, making it difficult for the lungs to inflate and not relieved by manoeuvres to clear the soft tissues, such as jaw-thrust, positive pressure ventilation up to 20 cmH2O and Guedel cannula(5) and *bronchial aspiration*: gastric contents in the upper airway, with or without aspiration pneumonitis.(6) |
| --- | --- |
| **Clinical data included in the model** | Age, recorded in completed years at the time of surgery; ASA physical status; lung or airway disease; symptoms of upper airway infection (URI) such as sneezing, runny nose, dry cough, productive cough, sore throat, fever, purulent nasal discharge, dyspnea; passive smoking at home, and prematurity (birth less than 37 weeks). |
| **Lung or airway affections** | Asthma or a history of wheezing, sleep apnea and hypopnea syndrome, bronchopulmonary dysplasia, mucopolysaccharidosis, bronchopneumonia, tracheal stenosis, pleural effusion, micrognathia, Pierre-Robin syndrome, Treacher-Collins syndrome, laryngomalacia, pharyngomalacia, tracheomalacia, cystic fibrosis, airway or lung tumours, pulmonary hypertension, tracheoesophageal fistula, pulmonary hypoplasia, and hyaline membrane disease. |
| **URI (upper-respiratory infection)** | Current (present at the moment of surgery) or recent (present during the last six weeks). |
| **Airway surgeries** | Labiaplasty, palatoplasty, tonsillectomy, adenoidectomy, rigid or flexible fiberoptic bronchoscopy, tracheoplasty, supraglottoplasty, tracheoscopy, tracheal dilation, tooth extraction, drainage of tonsil abscess, glossectomy, glossoplasty, excision, oral cavity lesion, and laryngoscopy with biopsy. |
| **Nature of the procedures** | Elective or non-elective (urgency/emergency) |
| **Intra-operative (anaesthetic) variables** | The airway device used (tracheal tube, supraglottic device, and face mask), type of anaesthetic induction (intravenous, pure inhalation or mixed), type of anaesthesia maintenance (inhalation or total venous), and use of neuromuscular blockers. |
