# Supplementary material for: Incidence and risk factors of perioperative respiratory adverse events in pediatric surgical patients: Development and validation of a predictive model in Brazil
Source: PLoS One. 2026 Apr 21;21(4):e0347477. doi: 10.1371/journal.pone.0347477 (PMC13098903; doi:10.1371/journal.pone.0347477)
Supplement: S2 Table — (DOCX) [file pone.0347477.s002.docx]

**Incidence and Risk Factors of Perioperative Respiratory Adverse Events in Pediatric Surgical Patients: Development and Validation of a Predictive Model in Brazil**

**Supporting information**

**S.2 Table** - Observed vs expected perioperative respiratory adverse events for deciles of risk.

| Positive if greater than or equal to | Sensitivity | 1 - Specificity |
| --- | --- | --- |
| .15 | **.66** | .31  (0.**68**) |
| .20 | **.49** | .21 (0.**78**) |
| .25 | **.30** | .09 (0.**90**) |
| .30 | **.27** | .07 (0.**92**) |
