# Supplementary material for: Incidence and risk factors of perioperative respiratory adverse events in pediatric surgical patients: Development and validation of a predictive model in Brazil
Source: PLoS One. 2026 Apr 21;21(4):e0347477. doi: 10.1371/journal.pone.0347477 (PMC13098903; doi:10.1371/journal.pone.0347477)
Supplement: S3 Table — Model 1 includes only the hospital variable. Model 2 including hospital variables and anesthetic and surgical variables. In model 3, those without statistical significance were excluded. (DOCX) [file pone.0347477.s003.docx]

**Incidence and Risk Factors of Perioperative Respiratory Adverse Events in Pediatric Surgical Patients: Development and Validation of a Predictive Model in Brazil**

**Supporting information**

**S.3 Table -** Logistic Regression Model for PRAE considering the hospital as a random effect. Model 1 includes only the hospital variable. Model 2 including hospital variables and anesthetic and surgical variables. In model 3, those without statistical significance were excluded. ASA-PS, American Society of Anesthesiologists physical status; OR, odds ratio; CI, confidence interval; URI, upper respiratory infection.

| **Variables** | **OR (CI95%) or variance** | ***p* value** |
| --- | --- | --- |
| **Model 1 (n = 1339)** |  |  |
| Hospital | Variance = 0.06346 | 1.89% of the residual variation in the propensity to have respiratory complications is attributable to unobserved hospital characteristics |
|  |  |  |
| **Model 2 (n = 1190)** |  |  |
| Hospital | Variance = 0 |  |
| Age < 1 y | 2.07 (1.34 – 3.21) | 0.009 |
| ASA-PS 1  ASA-PS 2  ASA-PS 3  ASA-PS 4 | Ref.  1.20 (0.76 – 1.89)  1.70 (1.04 – 2.79)  1.40 (0.33 – 5.83) | 0.434  0.035  0.648 |
| Obesity | 0.70 (0.28 – 1.76) | 0.452 |
| Lung or airway disease | 1.87 (1.24 – 2.84) | 0.003 |
| Current URI | 2.64 (1.33 – 5.25) | 0.006 |
| URI < 6 weeks | 1.47 (0.89 – 2.40) | 0.130 |
| Passive smoking | 1.07 (0.74 – 1.55) | 0.707 |
| Prematurity | 2.28 (1.55 – 3.38) | <0.001 |
| Airway surgery | 1.74 (1.08 – 2.80) | 0.023 |
| Urgency | 1.73 (1.12 – 2.66) | 0.013 |
| Endotracheal intubation | 1.62 (1.9 – 2.38) | 0.016 |
| Premedication | 1.07 (0.71 – 1.59) | 0.757 |
| Laryngotracheal topical anesthesia | 0.97 (0.56 – 1.67) | 0.907 |
| Endovenous induction | 0.86 (0.58 – 1.27) | 0.451 |
|  |  |  |
| **Model 3 (n = 1339)** |  |  |
| Hospital | Variance = 0 |  |
| Age < 1 y | 2.32 (1.59 – 3.39) | <0.001 |
| Lung or airway disease | 2.28 (1.63 – 3.18) | <0.001 |
| Current URI | 3.71 (2.06 – 6.66) | <0.001 |
| Prematurity | 2.56 (1.80 – 3.64) | <0.001 |
| Airway surgery | 1.38 (0.97 – 1.97) | 0.069 |
| Endotracheal intubation | 1.67 (1.17 – 2.39) | 0.005 |
