# Supplementary material for: Incidence and risk factors of perioperative respiratory adverse events in pediatric surgical patients: Development and validation of a predictive model in Brazil
Source: PLoS One. 2026 Apr 21;21(4):e0347477. doi: 10.1371/journal.pone.0347477 (PMC13098903; doi:10.1371/journal.pone.0347477)

**Incidence and Risk Factors of Perioperative Respiratory Adverse Events in Pediatric Surgical Patients: Development and Validation of a Predictive Model in Brazil**

**Supporting information**

**S.1 Figure –** The graph shows the prediction of instability for the original model and the 1000 bootstrap samples. In it we observe the dispersion of the predicted values ​​ 𝐵 (y-axis) for each individual in relation to their original predicted value (x-axis).


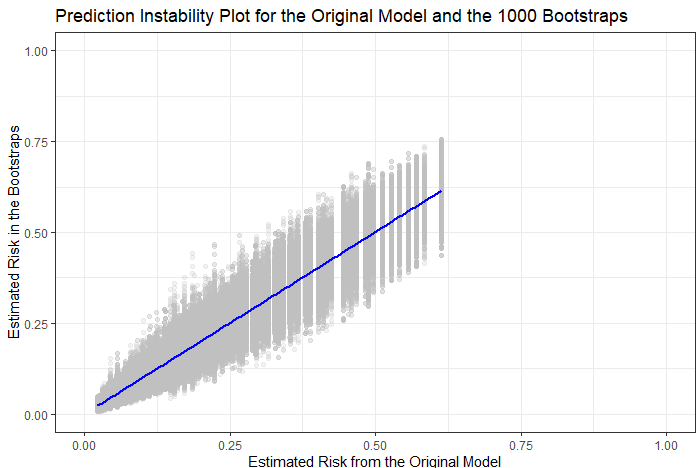

Supplement: S1 Fig — In it we observe the dispersion of the predicted values 𝐵 (y-axis) for each individual in relation to their original predicted value (x-axis). (DOCX) [file pone.0347477.s004.docx]
