# Supplementary material for: Incidence and risk factors of perioperative respiratory adverse events in pediatric surgical patients: Development and validation of a predictive model in Brazil
Source: PLoS One. 2026 Apr 21;21(4):e0347477. doi: 10.1371/journal.pone.0347477 (PMC13098903; doi:10.1371/journal.pone.0347477)

**Incidence and Risk Factors of Perioperative Respiratory Adverse Events in Pediatric Surgical Patients: Development and Validation of a Predictive Model in Brazil**

**Supporting information**

**S.2 Figure –** The calibration instability plot examines the instability in the calibration curves for the bootstrap models 𝐵 when evaluated on the original dataset. In it, the 𝐵 curves are overlaid on the same plot, along with the original calibration curve of the original model applied to the original data.


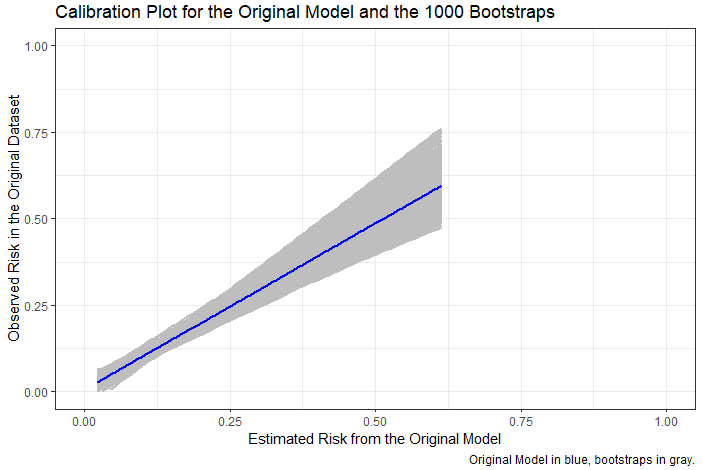

Supplement: S2 Fig — In it, the 𝐵 curves are overlaid on the same plot, along with the original calibration curve of the original model applied to the original data. (DOCX) [file pone.0347477.s005.docx]
