# Supplementary material for: Incidence and risk factors of perioperative respiratory adverse events in pediatric surgical patients: Development and validation of a predictive model in Brazil
Source: PLoS One. 2026 Apr 21;21(4):e0347477. doi: 10.1371/journal.pone.0347477 (PMC13098903; doi:10.1371/journal.pone.0347477)

**Incidence and Risk Factors of Perioperative Respiratory Adverse Events in Pediatric Surgical Patients: Development and Validation of a Predictive Model in Brazil**

**Supporting information**

**S.3 Figure -** APE can be shown graphically in a MAPE instability plot, which is a scatter of the MAPE value (y-axis) for each individual against their estimated risk from the original prediction model (x-axis). This plot reveals the range of MAPE values ​​and helps to identify if and where instability is of most concern for the original predictions.


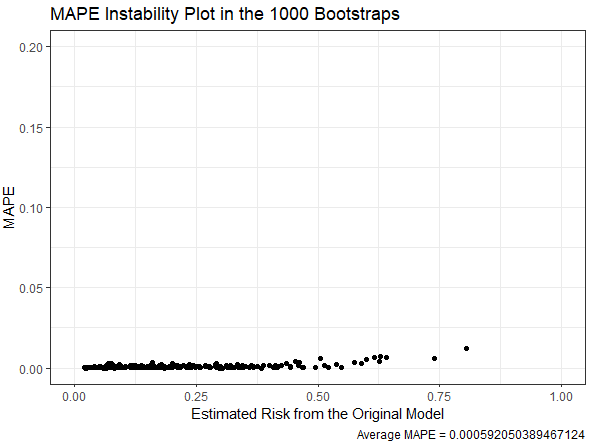

Supplement: S3 Fig — This plot reveals the range of MAPE values and helps to identify if and where instability is of most concern for the original predictions. (DOCX) [file pone.0347477.s006.docx]
